# Requirement of Simultaneous Assessment of Crystal- and Supernatant-Related Entomotoxic Activities of *Bacillus thuringiensis* Strains for Biocontrol-Product Development

**DOI:** 10.3390/toxins6051598

**Published:** 2014-05-20

**Authors:** Ronaldo Costa Argôlo-Filho, Robson Luz Costa, Daniele Heloisa Pinheiro, Fábio Mathias Corrêa, Fernando Hercos Valicente, Alan William Vilela Pomella, Leandro Lopes Loguercio

**Affiliations:** 1 Department of Biological Sciences (DCB), State University of Santa Cruz (UESC), Rod. BR 415, Km-16, Ilhéus-BA 45662-900, Brazil; E-Mail: leandro@uesc.br; 2 Farroupilha Laboratory, Av. Cica No. 555, Patos de Minas-MG 38706-420, Brazil; E-Mails: robsonluzcosta@yahoo.com.br (R.L.C.); alan@grupofarroupilha.com (A.W.V.P.); 3 Embrapa Maize and Sorghum, Rod. MG 424, Km 65, Sete Lagoas-MG 35701-970, Brazil; E-Mails: daniele.hp@hotmail.com (D.H.P.); fernando.valicente@embrapa.br (F.H.V.); 4 Department Exact and Technological Sciences (DCET), State University of Santa Cruz (UESC), Rod. BR 415, Km-16, Ilhéus-BA 45662-900, Brazil; E-Mail: fmcron@gmail.com

**Keywords:** protein secretion, feeding bioassays, beta-exotoxin, Cry, alternative *Bt* toxins, culture supernatant

## Abstract

Bioinsecticides with lower concentrations of endospores/crystals and without loss of efficiency are economically advantageous for pest biocontrol. In addition to Cry proteins, other *Bacillus thuringiensis* (*Bt*) toxins in culture supernatants (SN) have biocontrol potential (e.g., Vip3A, Cry1I, Sip1), whereas others are unwanted (β-exotoxins), as they display widespread toxicity across taxa. A strain simultaneously providing distinct toxin activities in crystals and SN would be desirable for bioinsecticides development; however, strains secreting β-exotoxins should be discarded, independently of other useful entomotoxins. Entomotoxicity of crystals and SN from a Brazilian *Bt tolworthi* strain (*Btt*01) was tested against *Spodoptera frugiperda* to assess the potential for biocontrol-product development based on more than one type of toxin/activity. Tests showed that 10^7^ endospores mL^−1^ caused >80% of larvae mortality, suggesting *Btt*01 may be used in similar concentrations as those of other *Bt*-based biopesticides. When it was applied to cornfields, a significant 60% reduction of larvae infestation was observed. However, bioassays with *Btt*01 SN revealed a thermostable toxic activity. Physicochemical characterization strongly suggests the presence of unwanted β-exotoxins, with isolate-specific temporal variation in its secretion. Knowledge of the temporal pattern of secretion/activity in culture for all forms of toxins produced by a single strain is required to both detect useful activities and avoid the potential lack of identification of undesirable toxins. These findings are discussed in the contexts of commercial *Bt* product development, advantages of multiple-activity strains, and care and handling recommended for large-scale fermentation systems.

## 1. Introduction

*Bacillus thuringiensis* (Berliner) (*Bt*) is an entomopathogenic bacterium found in many different environments, producing a variety of Cry proteins during sporulation that are selectively toxic to various insect pests [1,2,3]. Once ingested by the target insect, these proteins cause osmotic imbalance and intestinal paralysis which lead to inanition, sepsis and death [4]. These characteristics have been successfully explored in the past 60 years in the field of biological control of insect pests. However, due to the emergence of resistant pest populations along with the use of *Bt*-based insecticides, obtaining new *Bt* strains and/or toxins that are more effective and selective in the control of these insect races is a constant focus of research [5]. In addition to the most widely used Cry proteins, several other insecticide molecules are produced by *Bt*, such as Cyt (cytolytic), Vip (vegetative insecticidal protein), Sip (secreted insecticidal protein), phospholipase C and exochitinase [6,7,8,9]; these have also been studied for potential use in biocontrol. However, not all of these alternative *Bt* toxins can be used for pest control, as it is the case with β-exotoxin (Thuringiensin). This is a thermostable compound of low molecular weight (701 Da) that is analogous to adenine or uracil nucleotides [10,11], showing its toxic action by interfering with processes of DNA-dependent RNA polymerization [12]. Therefore, it has a wide spectrum of biological toxicity over a variety of non-target species, including mammals. Such a characteristic has led the World Health Organization to prohibit the use of β-exotoxin-producing *Bt* strains in bioinsecticide formulations. Genes that regulate the synthesis of β-exotoxins in *Bt* are located in plasmids that also encode some Cry proteins [10].

Brazil is the third largest producer of maize (*Zea mays* L.); its cultivated area is 14.33 million hectares and 2011/2012 production was approximately 58 million tons, making up 8% of world production [13]. The fall armyworm, *Spodoptera frugiperda* (J.E. Smith) (Lepidoptera: Noctuidae), is a major pest of maize in several countries, causing production losses of approximately 30%–50% [14]. For its relevance to the world’s corn market, devising economically and environmentally acceptable control methods for this pest is yet a research focus, for which integrated pest management (IPM) has offered the best approaches [15]. For many years, the major form of armyworm control has been the use of synthetic chemical insecticides, which are well known to have caused deleterious effects to ecosystems and toxicity to humans [16,17,18]. Several forms of biological control using natural predators, live microorganisms or their metabolites, plant extracts, and viruses have been developed in order to minimize this impact [1,19,20,21,22]. In this context, the use of *Bt*-based biopesticides is highlighted, because toxins of certain strains have successfully controlled fall armyworm, by either direct application or plant transformation [23,24,25]. Furthermore, only recently the tropical germplasm of *Bt* has started to be investigated for their potential in biocontrol of fall armyworm races, in the context of corn production in Brazil [26,27,28].

To assure economy and competitiveness for biopesticide production, the use of bacterial strains producing large quantities of toxins that are efficient at lower concentrations is desirable. In Brazil, *Bt* germplasm from tropical and temperate regions (e.g., HD series) have been described as being highly effective against fall armyworm. A tropical *Bt* strain, “*Btt*01,” has been characterized and identified as belonging to the subspecies *tolworthi*; it produces a bipyramidal crystal of δ-endotoxins and its LC_50_ was estimated to be ~8 × 10^6^ endospores mL^−1^ for fall armyworm [25]. This suggest the possibility of its use in similar concentrations to those of commercial products, especially the HD1-based biopesticides (LC_50_ of 6 × 10^6^ endospores mL^−1^) used to control this pest [29,30]. In addition, entomotoxic activities of proteinaceous nature that are secreted in its culture supernatant (SN) at the log phase have also been evaluated [28,31,32]. Taking this information into account, and aiming at improving the economic potential of exploring this strain for the biocontrol market, we evaluated entomotoxic activities against fall armyworm from pellet (crystals) and from SN. Such a possibility of having two distinct toxic activities from the same culture time in a large-scale fermentation system would certainly be technically and economically advantageous. Thus, using *Btt*01 as the experimental model, this study aimed to verify: (i) whether the use of the lowest possible initial endospore concentration of this strain is agronomically viable for fall armyworm biocontrol in the field; (ii) whether there is suitable entomotoxic activity for use in biocontrol-product development in culture SN collected at the same time that the endospores and crystals are obtained under industrial-scale fermentation; and (iii) whether temporal assessments of toxins synthesis/secretion during a *Bt* culture is relevant for decision-making processes regarding bioinsecticide production. Because an unequivocal detection of the undesired β-exotoxin was found in the *Btt*01 strain, the results taken together indicate that concomitant evaluations of both fractions containing toxins in *Bt* cultures (pellet and SN), coupled with assessment of their temporal synthesis/secretion during culture are fairly necessary for more efficient and environmentally safer large-scale production schemes involving promising isolates.

## 2. Results

### 2.1. Entomotoxicity of Btt01 δ-Endotoxins in Laboratory and Field Bioassays

Preliminary studies have shown that the *Btt*01 strain displays a higher δ-endotoxins activity against *S. frugiperda* than other *Bt* isolates [25]. In this study, we tested a standardized concentration of 10^7^ endospores mL^−1^ for this strain, which is similar to other commercial *Bt*-based products and preserves satisfactory biocontrol effects. Firstly, three different diet types (artificial, detached leaves and V4-stage maize plants) were assessed to verify whether they would affect the results of larvae mortality by the Cry toxins from the *Btt*01. The results showed that the tested concentration was efficient for larvae control, with an overall mortality rate of ~80%. Although there was no significant difference between diet types, the artificial one became the best option because of lesser experimental variation. The high overall mortality in all diet treatments indicated that they did not inactivate the toxins or prevent the larvae access to them (data not shown).

**Figure 1:**
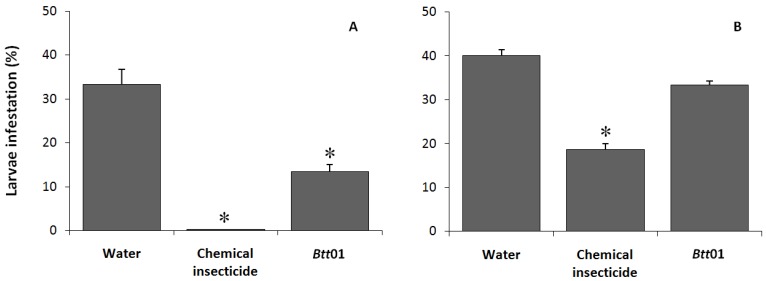
*Btt*01 Cry toxins insecticidal activity against *S. frugiperda* in (**A**) non-irrigated and (**B**) center-pivot irrigated maize fields. The synthetic insecticide Lannate BR^®^ (DuPont) was used as positive control. The infestation level was verified by the number of plants with characteristic signs of injury. The results show the means of 3 replications + std error. Statistical differences were determined by contingency 2 × 2 chi-square tests on the data from directly counting the number of larvae infestations (* *p* < 0.05).

After the feeding bioassays at laboratory scale have confirmed the entomocidal efficiency of this strain, field trials were carried out with the dual purpose of verifying whether this high toxicity was maintained under field conditions, and to assess the effects of irrigation on the efficiency of the toxins applied. Tests were performed under usual conditions of irrigation (center pivot system applying 3 mm water day^−1^) and insecticide applications on maize crops (Figure 1). Results from the non-irrigated maize showed that the application of *Btt*01 at 10^7^ endospores mL^−1^ was effective in controlling *S. frugiperda* infestation in the field. Larvae infestation decreased to less than half of negative control, and did not significantly differ from the synthetic insecticide effects (Figure 1A). On the other hand, effects of the *Btt*01 on the irrigated crop did not significantly reduce larvae infestation (Figure 1B). Comparing both cases by the Abbott’s formula [33], the efficiency of *Btt*01 Cry toxins in non-irrigated crop was 60%, but only 17% in irrigated maize, indicating a reduction of 71.7% in the biocontrol efficiency. Similarly though, the synthetic insecticide also showed a reduction in armyworm’s control efficiency, decreasing from 100% in non-irrigated maize to only 53% in the irrigated crop.

### 2.2. Entomotoxicity of Btt01 Supernatant

**Figure 2:**
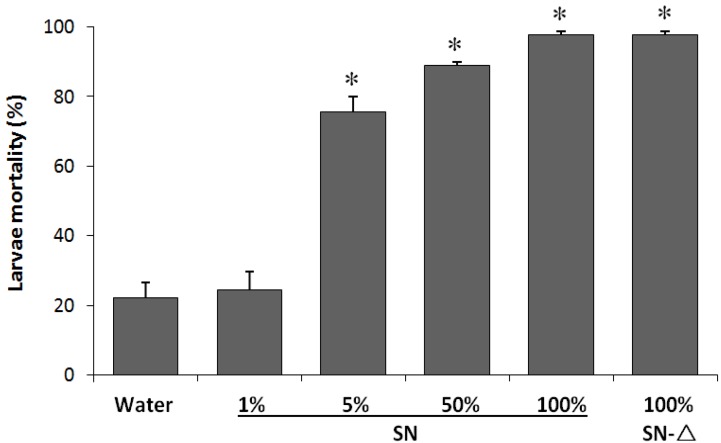
*Btt*01 supernatant toxic activity against *S. frugiperda*. Artificial diets were soaked in unheated (SN) and heated (SN-∆) supernatant. The SN-∆ treatment corresponds to heating in water bath at ~100 °C for 20 min. The percentage values at the x-axis refer to SN concentration in sterile water. The results are the mean of 3 replicates + std error. Statistical differences were determined by contingency 2 × 2 chi-square tests on data from directly counting the number of dead larvae (* *p* < 0.05).

Based on previous studies at laboratory scale [28,31,32], this strain has also shown some promise as a source of useful entomotoxic activity secreted in the SN. The possibility of using the SN of *Btt*01 as an alternative source of useful entomotoxins was thus verified by applying the residual liquid fraction of a large-scale fermentation, from the same time used to produce spores, in feeding bioassays. The results showed that toxicity of this SN against *S. frugiperda* was very high, even when a remarkable dilution to only 5% of the initial concentration was performed. Although it could have indicated desired results, the fact that such an activity was fully thermostable demonstrated otherwise; the heated SN at a high temperature showed identical levels of mortality (~98%) in relation to the non-heated treatment (Figure 2). Therefore, the whole activity observed for this SN was due to a non-proteinaceous form of toxin.

**Figure 3:**
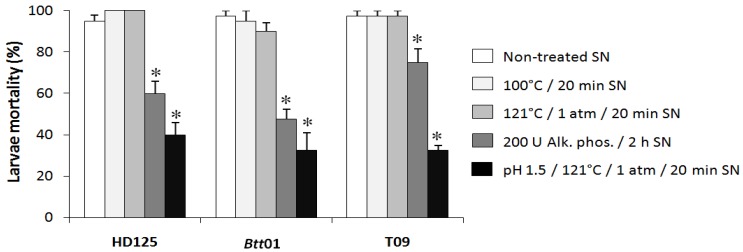
Feeding bioassay against *S. frugiperda* with different inactivation treatments for the thermostable toxin in the SN of three *Bt* strains. Artificial diets were soaked in SN treated as indicated in the legend on the right. The results are the mean of 4 replicates + std error. Statistical differences were determined by contingency 2 × 2 chi-square tests on data from directly counting the number of dead larvae (* *p* < 0.05).

Some *Bt* strains are producers of the non-protein, thermostable β-exotoxins [10], which show a large spectrum of toxicity across taxa. Two other *Btt* strains, HD125 and T09, derived from temperate climates (USA and France, respectively), are known to produce β-exotoxins [34,35]. In order to confirm whether the *Btt*01 strain from a tropical origin (Foz do Iguaçu-PR, Brazil) also produces this same type of toxin, an experiment was carried out to compare the toxic activities of these strains. Treatments known to eliminate the β-exotoxin toxic activity (see Experimental Section) were tested simultaneously with others that do not (Figure 3). Bioassays using *Btt*01 SN against *S. frugiperda* showed high mortality levels of the same magnitude of those displayed by HD125 and T09 strains, when treated only with high temperature. However, SN treatments based upon alkaline phosphatase digestion, or heating at low pH (<3.0) tend to inactivate this toxin [9,33]. The mortality results for all three strains with their SNs treated in these two ways confirmed a tendency of inactivation of the thermostable toxic activity. However, at least under the conditions employed in this study, a complete inactivation was not observed. Furthermore, a strain-specific inactivation effect for the alkaline phosphatase treatment was also observed (Figure 3). Taken together, these results indicated that the *Btt*01 tropical strain produces at least one type of β-exotoxin in its SN, under the same large-scale culture conditions used to produce the endospores suitable for biocontrol purposes.

**Figure 4:**
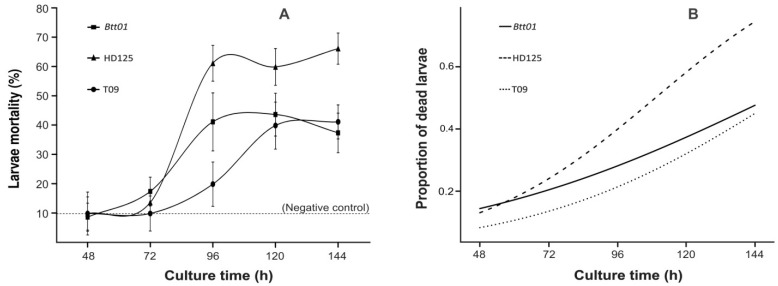
Temporal profile in culture of thermostable toxic activity against *S. frugiperda* from SN of three *Bt* strains. Cultures were performed at small-scale laboratory conditions. (**A**) Temporal profile of the number of dead larvae per isolate (given in %), per culture time. Dashed line corresponds to the average levels of mortality in the bioassay controls with only water application to the diets. The results indicate the means of 8 replicates + std error; (**B**) Generalized models of larvae mortality per isolate as a function of culture time, with a logistic regression. Statistical deviance analyses were performed for the variables “isolates” and “culture times” individually, as well as of their interaction; all results showed significance at *p* < 0.05 (see Experimental Section).

Differences in proteotoxin secretion among isolates during bacterial growth have shown to interfere with the final toxic effects of the respective SNs [28]. It was thus necessary to determine whether a similar temporal regulation also occurred with secretion of low-weight metabolites, such as the β-exotoxins. The existence and extent of this possible temporal variation, and how it would affect the insecticidal activity of the strains, were evaluated under small-scale (laboratory) culturing conditions. Despite being inoculated and cultured in the same way (see Experimental Section), the studied strains varied in relation to the time that production/detection of thermostable toxin first occurred, as well as to the highest mortality levels achieved by each one (Figure 4). HD125 and *Btt*01 began to show toxicity with ~72–80 h of incubation, rapidly reaching a peak at ~96–100 h, and remaining at similar levels afterwards. However, the mortality levels achieved by the HD125 were always greater than *Btt*01, with a tendency for another increase later on (144 h), whereas the latter showed a declining pattern (Figure 4A). In contrast, the T09 strain began to show toxicity only later, after ~90–96 h, and reached its maximum level at ~120 h, remaining so until the end. At the last two time points, mortality of this strain was very similar to the *Btt*01. After the 96-h time point, the HD125 showed higher levels of larvae mortality than others. In the experimental conditions used, the sporulation phase for the three strains started at ~72 h. Generalized modeling with a logistic regression showed statistical significance (*p* < 0.01) for the individual effects of the factors “isolates” and “culture time” and for their interaction (*p* < 0.05)(Figure 4B). For any given mortality proportion >0.18, the isolates differ in their timing in which thermostable toxicity is secreted to provide such mortality; for instance, a dead larvae proportion of 0.2 is achieved at ~65, 71 and 93 h of culture for HD125, *Btt*01 and T09, respectively.

## 3. Discussion

The three most relevant challenges that integrated insect-pest management (IPM) programs must overcome are (i) assurance of economic viability, efficiency, and environmental sustainability of the control measures; (ii) reduction or elimination of threats to environment and health; and (iii) prevention or delay of resistance development by the target pest [15,36,37]. The aspects to be considered for proper decision-making processes in this context include the use of single or multiple suitable isolates combined with distinct mechanisms of action, better formulations and improved delivery methods [38]. Furthermore, host-specificity is a key factor to determine whether biocontrol strategies will be efficient and environmentally safer [39,40]. Hence, alternatives of biocontrol agents and/or derived active substances are constantly needed, leading to the search for novel isolates carrying more effective toxins and/or new specificities. The work here reported is an example of a comprehensive sequence of steps required for a full characterization of a promising biocontrol *B. thuringiensis* (*Bt*) isolate, such that both its advantageous aspects and drawbacks are discussed in the context of a most appropriate management of the production system.

In the context of *Bt* applications, a technically and economically desirable condition is to have formulations with lower amounts of endospores that keep the same efficiency of the final product. The *Btt*01 δ-endotoxins used in this study were highly toxic and effective against fall armyworm (*S. frugiperda*) at a concentration of endospores very similar to other *Bt*-based biocontrol products available in the market. A possible explanation for this strain efficiency would be its specific constitution of *cry* genes. *Btt*01 carries the *cry1Ab, cry1B, cry1E* and *cry1Fb* genes and their proteins form a bipyramidal crystal [25]. The Cry1 group has been described as effective against the genus *Spodoptera*, whereas Cry1B and Cry1F are among the most potent proteotoxins already found [41,42,43]. Moreover, previous studies had shown clear advantages for this specific composition of Cry proteins (including Cry1E) on the toxicity to *S. frugiperda* under tropical conditions [26]. Therefore, on the basis of preliminary studies [26,44] and on the laboratory and field bioassays of this work, the *Btt*01 strain showed a great potential for use in biocontrol applications, mostly in the form of biopesticides based on parasporal inclusions of proteinaceous (hence thermolabile) nature.

Most of the screening studies reported in the literature tend to focus on laboratory assays, which do not allow observation of environmental factors affecting the *Bt* physiology or toxins. An evidence for a washing out interference of crop-applied water on the insecticidal action was observed in the field experiments (Figure 1): *Btt* endospores were more efficient in non-irrigated maize, even with the irrigation occurring at least 24 h after endospore application. Moreover, irrigation also decreased the synthetic chemical insecticide efficiency, although to a lesser extent, possibly due to a faster entomocidal action by contact. Since the synthetic insecticides are usually recommended to be used only after appearance of the first signs of insect damage, producers generally have the habit of considering the use of biocontrol products in the same way [45]. However, as general guidelines for a better efficiency of these products (confirmed in this study), bioinsecticide applications must reach the initial stages of insect development that are more susceptible [46], occurring before plant damages are visible. In any case, the control efficiency of *Btt*01, under our experimental conditions, was not too different from that of the synthetic insecticide, which suggests that simple management schemes that coordinate *Bt* applications (at proper pest stages) and irrigation may be sufficient to compensate for the difference between the two methods.

Because several other toxins have shown to be secreted in the *Bt* culture SNs (Vip, Cry1I, Sip, exochitinase, α- and β-exotoxins [9,10,11,47]), and because several cases of development of insect resistance to Cry toxins have already been described [4,48], the search for new biological pest control alternatives has been frequent. In this context, confirming toxic effects of a SN obtained from the same large-scale fermentation stage and conditions that are used for commercial production of Crys would be two-fold advantageous: it could provide useful alternative toxins, as well as improve the economics of the production system by exploring more than one type of toxin simultaneously. However, an analysis of SN toxicity of *Btt*01 was important in a different sense, *i.e.*, for a better understanding of potential limitations in *Btt*’s physiology. In previous studies, the protein fraction of *Btt*01 SN have been isolated, concentrated and tested against fall armyworm [31,32]; since no relevant activity had been found, the toxic SN activity here observed (Figure 2) is likely not related to any proteotoxin. It is strongly suggested that the presence of a thermostable and highly toxic activity against *S. frugiperda* (Figure 2) was due to the undesired β-exotoxin, because it could be inactivated by phosphate ester cleavage or by high temperature treatment (autoclaving) at a very acidic pH [11,33] (Figure 3). 

Thinking on alternative methods to detect β-exotoxins, a correlation between their production and the presence of *cry1B* gene has been suggested [49], which could interestingly allow indirect detection of those toxins through easy-to-handle molecular techniques, such as PCR. However, despite this gene being found in *Btt*01 [25,48], parallel studies in other *Bt* isolates (tropical germplasm) showed that ~50% of them carried the same gene, but did not present thermostable toxic activities at any point in their growth, even after the sporulation phase (not shown). This therefore suggests that PCR-based detection of specific *cry1B* aiming at identifying β-exotoxin-producing strains is not a useful technique. In terms of the thermostable toxic SN activities, it is noteworthy that the maximum levels of mortality shown in Figure 4 for *Btt*01 and the other strains were different than those from experiments with industrial-scale SN (Figure 2). Possible reasons for this discrepancy could be differences in the SN collection times, culture volumes (1500 L *vs.* 50 mL), inoculum conditions, and general culture conditions (industrial fermenter with aeration and pH control *vs.* culture bottles with orbital shaking and without pH control). In this context, it is worth mentioning that, for the *Btt*01 strain, endospores are produced after 24 h in large-scale fermentation (see Experimental section), but only after ~72 h in small-scale culture conditions (Figure 4). In addition, the significantly higher entomotoxic activities observed for strain HD125 (Figure 4) could have been due to greater levels of toxin secretion, or to a different, more toxic type of β-exotoxin. Hence, culturing experiments in industrial fermenters that compare the timing of thermostable entomotoxin secretion among the tested strains are warranted to verify if their relative temporal patterns of β-exotoxin secretion (see below) are the same or not.

Due to an obvious practical facility and convenience, screening studies of large collections of microorganisms usually assess a number of isolates simultaneously, which frequently implies the use of the same culture time or same optical density (OD) for detection of their activity of interest. However, comparison of entomotoxic activities for different *Bt* strains obtained under these circumstances are intrinsically compromised by physiological variations observed among the isolates [28]. The concomitant collection of SNs in a same given time or OD can display toxin secretion with strain-specific variation, even when all had the same amount of cells at inoculation [28]. This may generate temporally distinct toxicity profiles for each strain, and consequently, potential misclassification of promising isolates (mainly if they are evaluated for toxicity in culturing times that are not the most favorable for identification of the toxic activities of interest). Interestingly, this same isolate-specific temporal effect was also observed in the secretion of thermostable β-exotoxins in our study (Figure 4). Therefore, such temporal and strain-specific variations in the production/secretion of toxins (of any nature) suggest that screening processes of *Bt* collections will only be effective to determine the most promising strains if sufficient knowledge of their physiology in different culture conditions over time is achieved. Taken together with the aspects discussed above, this information increases even further the technical capability to handle promising isolates for given toxins that also produce other undesired compounds. In the case of the strain *Btt*01, for instance, this information now makes it possible to constitute commercial preparations of endospores in a proper manner, by avoiding the culture moment in which the undesired toxin starts being secreted and/or by properly treating it before disposal.

Although β-exotoxin is highly toxic against insects, it also causes deleterious effects on non-target organisms, including mammals, because it is a nucleotide analog that affects RNA synthesis [11,12,50]. Because of this health and environmental threat, the World Health Organization has prohibited the use of *Bt* strains producing this toxin in bioinsecticide formulations [50], though some countries still use β-exotoxin products in specific control programs of flies that are resistant to other insecticides [51]. Considering the positive results of this study in relation to the potential use of this tropical strain in biocontrol strategies, based on its δ-endotoxins composition [25,26,44] and action in the field, it is here postulated that the assessment of *Bt* collections also for the presence of β-exotoxins appears to be a necessary routine in research labs and companies producing *Bt*-based bioinsecticides. One can, for example, include tests with SN-heated aliquots [28,31,32] to assess for entomotoxicity and thermostability; once the latter is detected, further inactivation-based characterization such as those employed here can be used. We argue that performing studies/verifications similar to these (including temporal assessments in culture) are not so difficult or expensive, and can provide a better understanding about the physiology of a given strain of interest, not only in relation to its toxins’ arsenal, but also with regards to which culture conditions they are produced. The possibility of better decision-making processes about treatment or disposal of strains/toxins may allow more detailed analysis of benefits–costs ratios and can render more efficient strategies for production and marketing of biopesticides.

## 4. Experimental Section

### 4.1. Bacillus Thuringiensis Strain and Culture Conditions

A soil-derived *B. thuringiensis* var. *tolworthi* strain, labeled as “*Btt*01”, originating from Foz do Iguaçu city (Paraná, Brazil), and belonging to the microbial bank of the National Maize and Sorghum Research Center (EMBRAPA-CNPMS), was used in this study. Two milliliters of *Btt*01 from a −80 °C stock were inoculated on 2 L of LB medium enriched with salts [31], and incubated under constant agitation of 200 rpm and at 28 °C, until reaching a concentration of ~10^9^ cells mL^−1^ by hemocytometer. This constituted a pre-inoculum culture for the subsequent large-scale fermentation. The total volume of this pre-inoculum (2 L) was added to a cylindrical industrial fermenter, containing 1500 L of the same medium. Culture was performed at 30 °C, pH 7.5 and 150 rpm, until reaching the concentration of ~10^9^ endospores mL^−1^, which occurs between 22 and 24 h of culture. After fermentation, the endospores were separated from the supernatant by a filtration module Microdyn^®^, with 0.2 mm polypropylene membrane under 20 psi for 8 h, resulting in a concentration of 2–3 × 10^10^ endospores mL^−1^.

### 4.2. Feeding Bioassays with Endospores of Btt01 against Spodoptera frugiperda

A first feeding bioassay based on Cry toxins of *Btt*01 was performed on artificial diet [31], using a concentration of 10^7^ endospores mL^−1^, previously estimated as sufficiently efficient against fall armyworm [25]. Twenty-five artificial diets of 1 cm^3^ each were soaked for 1 min in 5 mL of the above suspension, let dry for 30 min, and placed individually in 50 mL plastic containers. Two-day-old *S. frugiperda* larvae were added (one larva per diet cube, per container), and the containers were closed with transparent plastic lids [32]. The insects were kept at 25–28 °C, in a photoperiod of 12 h:12 h (light:dark), with the mortality assessed after 5 days of incubation. For the control treatment, diets were soaked in sterile distilled water.

A second bioassay was performed as described above, with corn leaves (from plants at the V4 stage) that were cut to 5 cm in length to replace the artificial diets. Tween 20^®^ (Merck) was added to spore suspensions at 0.05% (*v*/*v*), to ensure that the toxins were homogeneously spread over the leaves. Three leaves and one larva were placed in each 50 mL plastic container covered with a transparent lid.

The third bioassay with the Cry proteins was carried out using entire corn plants at the V4 stage, which were obtained from corn seeds previously sowed in ~200 mL of soil placed in 300 mL plastic containers (three plants per container). A volume of 7 mL of a 10^7^ *Btt* endospores mL^−1^ suspension and 0.05% (*v*/*v*) Tween 20^®^ were sprayed on the V4 plants, covering the entire surface of leaves, and let dry for 30 min. Fifteen 2-days old *S. frugiperda* larvae were added to each container. Care was taken to avoid contact of leaves between different containers to prevent larvae escaping. To monitor for levels of such escaping, adhesive tapes were placed around the edge of the container, with the sticky side facing inwards; the escaping detected was negligible. The containers were kept at 25–28 °C with a photoperiod of 12 h:12 h (light:dark), and the mortality was assessed after 5 days. For the control treatment, the plants were sprayed with sterile distilled water containing 0.05% (*v*/*v*) Tween 20^®^. All treatments were replicated three times for each of the three bioassays.

### 4.3. Entomotoxic Activity of Btt01 Endospores in the Field

Tween 20^®^ (0.05%) was added to a suspension of ~10^7^ endospores mL^−1^ for application in the field. This suspension was applied by a CO_2_ backpack sprayer (spray bar with five flat-fan 110-03 nozzles), at a volume of 200 L·ha^−1^. The applications were made after 3:00 pm in two maize-planted areas. The first area corresponded to a non-irrigated cornfield with plants at the V7 stage. Climatic conditions during the field evaluation of this treatment were average 22 °C temperature, lack of rainfall, and a photoperiod of ~13:11 h (light:dark). At the moment of endospores application, this area showed natural infestation by *S. frugiperda* with ~33% of plants displaying injury signs. The second area corresponded to irrigated maize by a center pivot, with an average of 20 mm water applied per week. The plants were also at the V7 stage, and the area showed ~40% of plants displaying larvae injury signs. As negative control, only water with 0.05% (*v/v*) Tween 20^®^ was sprayed on both areas. As positive control, 600 mL·ha^−1^ (=129 g·ha ^−1^) of methomyl insecticide (BR Lannate^®^, DuPont) were applied, following the manufacturer’s instructions. The plots were 4 m wide, 0.8 m spacing between rows of maize, and 25 and 50 m in length for non-irrigated and irrigated fields, respectively. The experimental design was randomized blocks with three treatments and three replicates. In addition, three applications were performed with a week’s interval between them. The evaluation of the experiment was done one week after the third application, counting infestation in 15 plants of central lines in each block for non-irrigated, and in 25 plants for irrigated maize. The presence of live insects was verified by typical features, such as insect feces, scratched leaves, laid eggs, and the direct view of moving larvae or adults [52]. The counts of infested plants were transformed into infestation percentage, averaging the three replicates. These percentages were compared to those infestations obtained in the control. The efficiency was calculated using Abbott’s formula, E(%) = (C − T)/C × 100, where E = percentage of efficiency; C = number of alive insects in the control; and T = number of alive insects in the treatment [53].

### 4.4. Feeding Bioassays with Supernatant of Btt01 against S. frugiperda

The supernatant (SN) from *Btt*01 culture in industrial fermenter, resulting from filtration system Microdyn (see above) and free of vegetative cells, endospores and crystals, was also tested for its toxicity against fall armyworm. In order to distinguish possible toxic activities between heat-sensitive and thermostable molecules, this SN was divided into two parts, with one part heated for 20 min in a water bath at 95 °C [10]. In addition, the unheated SN was also diluted with sterile distilled water at 1:1, 1:20 and 1:100 proportions. The toxicity of all these treatments (three replicates per treatment) was assessed in bioassays against *S. frugiperda* on artificial diet, using the same general conditions for diets preparation, feeding and evaluation times described above.

Since highly thermostable toxic activities were detected in the above experiments, tests for inactivation of these activities were performed based on digestion of SN with alkaline phosphatase [33], and on temperature- and pH-dependent hydrolysis of SN-containing phosphate–ester bonds [11]. These tests were carried out to determine whether such a toxic thermostable activity was due to the presence of β-exotoxins. Besides the *Btt*01, two other strains previously known to produce thermostable β-exotoxins, but from different genetic backgrounds, were used as positive controls: the HD125 (U.S. Department of Agriculture, Agricultural Research Services, Peoria, IL, USA) and T09 (Institute Pasteur, Paris, France) strains [34,35]. From pre-cultures set up as described above, cultures for the three strains were established in 50 mL LB broth in 250 mL flasks, with an initial concentration of 10^6^ cells mL^−1^. These cultures were incubated at 28 °C under constant agitation of 200 rpm for 96 h. The SN was then separated from the biomass by centrifugation at 13,000 × *g* for 10 min. Half of the total SN volume was taken for toxin inactivation test by alkaline phosphatase: the SN pH was raised to 9.0 by addition of 2 N NaOH for better enzyme activity; alkaline phosphatase was added for 2 h in a 37 °C water bath, in four aliquots of 25 U every 30 min [33]. The other half of SN volume was taken for toxin inactivation test by phosphate–ester hydrolysis mediated by pH. In this case, the SN pH was reduced to 1.5 by addition of HCl (37%), and then autoclaved for 20 min at 121 °C and 1 atm [11]. Following these steps of possible deactivation of thermostable activity (which would confirm the β-exotoxin nature of the SN insecticidal activity), these SN treatments (four replicates each) were applied to the same feeding bioassays against *S. frugiperda* on artificial diets.

Possible temporal variations in β-exotoxin detection among these three strains were verified by evaluating their SNs collected at different culture times. From −80 °C stocks, these strains were set to grow in 50 mL flasks containing 10 mL of LB broth supplemented with salts [31], in the same overall conditions described for the pre-cultures (see above). After 12 h of growth, the number of cells was verified by hemocytometer. Volumes from these pre-cultures for each strain were adjusted to provide inocula at a same initial concentration of 10^6^ cells mL^−1^, in 50 mL cultures that were set and incubated as described above. For each strain, one 50 mL culture flask was set for each SN sampling at 48, 72, 96, 120 and 144 h of culture, respectively (total of five 50 mL flasks per strain). After each of these times, the corresponding culture was centrifuged at 13,000 × *g* for 10 min. The respective SNs were then carefully filtered through 3MM^®^ filter paper and autoclaved (121 °C and 1 atm for 20 min) prior to use in feeding bioassays against *S. frugiperda* (described above). Each isolate/time treatment was replicated 8 times. Time-collection treatments in which thermostable entomotoxic activity of SNs started to be detected indicate sufficient levels of β-exotoxin secretion.

### 4.5. Statistical Analysis

Considering the categorical type of variables and the counting nature of data for field (Figure 1), industrial fermentation supernatants (Figure 2) and β-exotoxins inactivation (Figure 3) experiments, chi-square analyses were employed on 2 × 2 contingency tables, comparing two treatments at a time (for Figure 2, the tests were performed only between individual % SN and the water control). The goodness-of-fit tests for the hypothesis of no difference in dead/alive larvae between treatments considered a significance level of 5%. The data from the temporal profiles of larvae mortality per isolates (Figure 4) were analyzed by generalized linear modeling with a binomial distribution and “logit” (logistic regression) link function to determine the difference between the proportions of live and dead insects in all performed “isolates” × “time-points” bioassays. Proportions were compared with a confidence level of 95% using the “glht” function of the “multcomp” library of the R statistical platform (R.2.15.1. version).

## 5. Conclusions

The results of this study, taken together, demonstrate that a single *Bt* strain can harbor and express different forms of toxins, which likely act in nature to help these bacteria live up to their characteristic as a *bona fide* pathogen of insect species [3,54]; this characteristic is advantageous in agricultural and biological control contexts if more than one useful type of toxin can be found for a single strain (e.g., Crys + Vips [28,32]). On the other hand, expression of multiple kinds of toxins, mainly when not all of them show beneficial features to non-target organisms (including humans), does require a deeper assessment of the genetics and physiology of each strain/isolate under investigation, as demonstrated by the case here reported. The key finding of this study is that a simultaneous analysis of pellet- and SN-related (thermolabile and thermostable) activities in *Bt* strains at different culturing times provides a more thorough and comprehensive understanding of their actual potential for biocontrol products manufacturing. This will certainly allow better-informed decision-making processes regarding economic viability of bioinsecticide production and employment of biological control strategies in any given agricultural system. We hope the approach here undertaken, as well as the ideas and considerations made, also become useful in other agricultural settings that deal with biological control programs, to fulfill the necessity of devising efficient strategies for food production with environmental sustainability.
